# In Vivo Imaging of Blood-Brain Barrier Leakage Using a Contrast Agent in Patients With Cerebral Amyloid Angiopathy: An Exploratory Study

**DOI:** 10.1212/WNL.0000000000214336

**Published:** 2025-11-05

**Authors:** Hilde van den Brink, Mariel G. Kozberg, Nazanin Makkinejad, John E. Kirsch, Michael J. Thrippleton, Thijs W. van Harten, Sabine Voigt, Whitney M. Freeze, Matthias J.P. Van Osch, Anand Viswanathan, Susanne J. van Veluw

**Affiliations:** 1 Department of Neurology, Massachusetts General Hospital, Harvard Medical School, Boston;; 2 Athinoula A. Martinos Center, Massachusetts General Hospital, Charlestown;; 3 Centre for Clinical Brain Sciences, University of Edinburgh, United Kingdom;; 4 Department of Neurology, Leiden University Medical Center, the Netherlands;; 5 Department of Radiology, Leiden University Medical Center, the Netherlands;; 6 UK Dementia Research Institute – Centre for Vascular Dementia Research, University of Edinburgh, United Kingdom.

## Abstract

**Background and Objectives:**

Blood-brain barrier (BBB) leakage may be an early step in the pathophysiology of cerebral amyloid angiopathy (CAA), possibly preceding hemorrhages. This exploratory study measured BBB leakage in vivo at the level of the leptomeningeal and small parenchymal vessels in patients with probable CAA. We hypothesized that BBB leakage from leptomeningeal and cortical small vessels would be higher in patients with CAA compared with controls and that leakage would be associated with hemorrhagic manifestations of CAA, that is, cortical superficial siderosis (cSS) and lobar cerebral microbleeds (CMBs).

**Methods:**

Patients with probable CAA without previous intracerebral hemorrhage and non-CAA patients with mild cognitive impairment from the memory clinic were recruited in this prospective observational exploratory study. Participants underwent 3T brain MRI with injection of a gadolinium-based contrast agent (Dotarem). Leakage from leptomeningeal vessels was assessed on postcontrast vs precontrast T2-fluid–attenuated inversion recovery as either focal or sulcal CSF enhancements. Dynamic contrast-enhanced scans were analyzed to quantify permeability-surface area product (PS): a measure of leakage from parenchymal small vessels.

**Results:**

Fourteen patients with CAA (mean age 67.7 ± 9.0 years; 57% female) and 7 non-CAA patients with mild cognitive impairment (mean age 70.1 ± 6.5 years; 29% female) were recruited. Focal CSF enhancements were observed similarly often in patients with CAA (7 [50%]) and non-CAA controls (4 [57%], *p* = 0.98), whereas sulcal CSF enhancements were only seen in patients with CAA (5 [36%] vs 0 [0%]). In patients with CAA, focal and sulcal CSF enhancement counts were associated with higher cSS volume (*B* = 2.61, *p* = 0.003; *B* = 1.02, *p* = 0.02), but not with CMBs. PS was numerically higher in the cortex in patients with CAA (5.08 ± 4.02 × 10^−4^ min^−1^) than in non-CAA controls (1.29 ± 4.08 × 10^−4^ min^−1^, *p* = 0.07), but it was not associated with CMB count or cSS volume (*p* > 0.67).

**Discussion:**

Leakage of gadolinium-based contrast agent through the BBB can be measured in vivo in CAA from the leptomeningeal vessels, and findings point to likely leakage from cortical small vessels as well. Leakage from leptomeningeal vessels was associated with cSS. Studies with follow-up data need to determine whether these measures could serve as a predictive biomarker in CAA.

## Introduction

Cerebral amyloid angiopathy (CAA) is neuropathologically defined by the deposition of β-amyloid (Aβ) in the walls of leptomeningeal vessels and small arteries, arterioles, and capillaries in the cortex.^1^ Clinically, CAA is a leading cause of lobar intracerebral hemorrhage (ICH), which is associated with high mortality rates and disability.^2^ Although an overall pathophysiologic framework has recently been proposed, the precise mechanisms that precede hemorrhage remain incompletely understood.^3^ Early blood-brain barrier (BBB) leakage may play a role in this.^4,5^ The BBB is formed by endothelial cells, which are held together by tight junctions and supported by astrocytes, pericytes, and extracellular matrix components.^6^ The BBB regulates molecular exchange between the blood and the CSF at the leptomeningeal level and between the blood and the parenchyma at the level of perforating arterioles, capillaries, and venules.^6^ The leakage of blood plasma proteins through the BBB into the brain and surrounding fluid-filled spaces may be a sign of early vessel wall breakdown in CAA before larger disruptions of the BBB occur in the form of hemorrhage (i.e., ICH, cerebral microbleeds [CMBs], and cortical superficial siderosis [cSS]).

In postmortem brain tissue of individuals with CAA, circulating plasma proteins fibrin(ogen) and immunoglobin G (IgG) have been observed in the walls of blood vessels without hemorrhage,^7,8^ indicative of a leaky BBB. Interestingly, fibrin deposition was observed both in vessel walls with circumferential as well as partial Aβ deposition,^9^ suggesting that BBB leakage may indeed be an early player in CAA pathophysiology. The leakage of blood substances into the parenchyma could trigger perivascular inflammation, which has been proposed to play a role in vessel remodeling and possibly in subsequent hemorrhage.^10^ Because of these ex vivo observations, we aimed to measure BBB leakage in vivo in patients with CAA. Specifically, we recruited patients with CAA without a history of ICH. As such, this study aimed to explore whether BBB leakage can be measured in presumed earlier pathophysiologic stages, before debilitating hemorrhages occur and, more importantly, when such hemorrhages could possibly still be prevented.

In vivo, BBB leakage can be measured with contrast-enhanced MRI.^11,12^ We measured extravasation of gadolinium-based contrast agent with 2 different imaging techniques that target leakage from different vessel populations. First, we studied CSF enhancement on heavily T2-weighted postcontrast fluid-attenuated inversion recovery (FLAIR) images. With postcontrast FLAIR imaging, leakage of gadolinium from the leptomeningeal vessels is visible as hyperintense signal within the CSF because of T1-relaxation time shortening by the extravasated contrast agent.^11,13,14^ Second, we used dynamic contrast-enhanced (DCE) MRI to measure gadolinium leakage from small vessels into the brain parenchyma. With this method, subtle gadolinium leakage into the extracellular-extravascular space can be assessed quantitatively as the permeability-surface area product, PS.^12^ We hypothesized that BBB leakage from both leptomeningeal and cortical small vessels is higher in patients with CAA compared with controls and that leakage is associated with hemorrhagic manifestations of CAA, that is, cSS and lobar CMBs.

## Methods

### Study Design and Participants

Participants were recruited from an ongoing single-center prospective memory-clinic cohort study at Massachusetts General Hospital. The parent study recruits nondemented participants of age 55 years or older with subjective cognitive complaints or mild cognitive impairment (MCI). Exclusion criteria were a history of head trauma, ischemic stroke, vasculitis or ICH, genetic CAA, and existing contraindications to MRI. From this memory-clinic cohort, we prospectively recruited participants with CAA and non-CAA control participants—with subjective cognitive complaints or actual MCI—for the current substudy. Our rationale was that by including a comparison group with overlapping clinical features, any group differences found in this study are likely more specific to CAA, rather than a general feature in the context of cognitive impairment. We recruited 14 patients with probable CAA and 7 non-CAA controls from the parent study to take part in the substudy and performed BBB imaging between August 2021 and September 2023. Probable CAA was determined based on the Boston criteria version 1.5,^15^ which means that all recruited patients with CAA also have a clinical diagnosis of probable CAA according to the Boston criteria version 2.0.^16^ We excluded patients with a history of CAA-related inflammation (N = 0) and because of safety guidelines for gadolinium injection, participants with chronic kidney disease (N = 2, 1 male and 1 female non-CAA patients) or abnormal renal function (N = 2, 2 male patients with CAA) were excluded from the substudy (i.e., estimated glomerular filtration rate [eGFR] <60 mL/min/1.73 mm^2^; both determined through medical record screening). One patient (female) with CAA was excluded because of an MR unsafe implant.

For this substudy, all participants underwent a baseline MR imaging session to assess BBB leakage. At the day of the scan, participants underwent a blood draw to measure eGFR to be able to correct for possible differences in renal function between patients with CAA and non-CAA controls that may influence gadolinium clearance. In addition, a subset of participants (8 patients with CAA and 2 non-CAA controls) underwent optional follow-up scans at 3, 6, and 24 hours to explore the evolution or delayed visibility of CSF enhancements over time. Scores on the Mini-Mental State Examination were taken from visits for the parent study that occurred within 6 months of participation in the substudy.

### Standard Protocol Approvals, Registrations, and Patient Consents

The study was approved by the Massachusetts General Hospital institutional review board (IRB protocol number: 2015P000145) and conducted in accordance with the Declaration of Helsinki. Informed consent was obtained from all participants before any study procedures.

### MRI Protocol

Images were acquired using a 3T Siemens Magnetom Skyra scanner with a 32-channel headcoil (Siemens Healthcare). The imaging parameters for each scan are reported in eTable 1. The protocol included a 3D T1-weighted scan (for brain segmentation and assessment of MRI-visible perivascular spaces and cortical cerebral microinfarcts), precontrast FLAIR (for the assessment of white matter hyperintensities [WMH] and cortical cerebral microinfarcts), susceptibility-weighted imaging (SWI) (for assessment of CMBs and cSS), and diffusion-weighted imaging (DWI) (for assessment of recent small subcortical infarcts).^17^

A 16-minute DCE scan was performed to assess BBB leakage from small vessels in the parenchyma. During this scan, a gadolinium-based contrast agent (Dotarem; Guerbet North America, Princeton, NJ) was administered intravenously at the start of the sixth volume (0.2 mL/kg body weight) at an injection rate of 3.5 mL/s, followed by a 15 mL saline flush at the same rate. For the DCE data analysis, B1 maps and T1 maps based on multiple flip angle (2°, 5°, and 12°) data were acquired (eTable 1).

A postcontrast FLAIR scan was also performed. The postcontrast FLAIR was used to assess CSF enhancements after the gadolinium injection. The time between gadolinium injection and postcontrast FLAIR imaging was about 22 minutes.

Finally, for the subset of participants who came back for optional follow-up scans at 3, 6, and 24 hours, the FLAIR scans were repeated to explore the evolution of CSF enhancements over time.

### Imaging Analysis

#### Neuroimaging Markers of CAA

MRI-visible perivascular spaces in the white matter centrum semiovale and basal ganglia were manually rated according to a 4-point ordinal scale on the T1-weighted scan (rather than a T2-weighted scan as per the STRIVE criteria, which was unavailable in the protocol).^18^ The presence or absence of WMH in a subcortical multispot pattern was determined based on the precontrast FLAIR scan,^19^ and cSS presence was rated on SWI. Cortical cerebral microinfarcts were manually rated based on the T1-weighted, precontrast FLAIR, and SWI scans, and recent small subcortical infarcts were rated on the DWI.^17^ WMH segmentation was performed with Samseg from FreeSurfer^20^ based on the T1-weighted and precontrast FLAIR scans. WMH segmentations were manually corrected where necessary before WMH volumes were calculated. WMH volumes were corrected for brain volume, which was determined by the segmentation of the T1-weighted scan with SynthSeg version 2.0 from FreeSurfer.^21^ CMB counting (on SWI) was supported by semiautomated rating software which automatically identifies possible lesions based on their spherical shape, after which identified candidates were manually checked to select CMBs.^22^ cSS volume (on SWI) was segmented with support from a semiautomated tool. In short, this tool identifies hypointense tubular structures in the 2D axial plane, and with the help of seed points and a 3D-growing region algorithm in the cSS region, a cSS mask can be generated.^23^ This mask was manually adjusted for false positives, and the cSS volume was calculated and corrected for brain volume.

All lesion ratings and segmentations were performed by rater H.v.d.B. who was blinded for the participant group. All ratings from H.v.d.B. were used for the analyses. To calculate the interrater agreement of H.v.d.B. with other raters, a subset of 8 participants were additionally rated by either S.v.V. or M.K. Interrater reliability was rated good to excellent (eTable 2).

#### CSF Enhancement Rating

**Figure 1:**
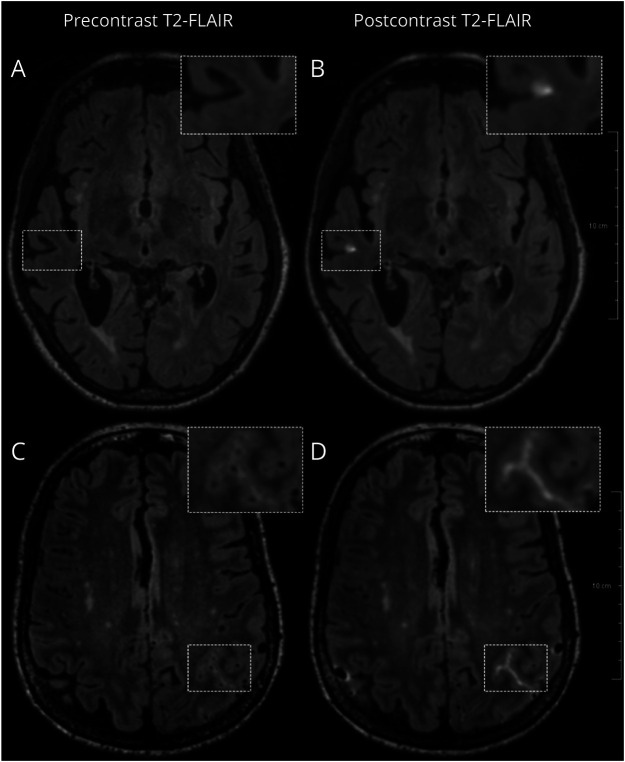
An Example of Focal (A and B) and Sulcal (C and D) CSF Enhancement in 2 Participants With Probable CAA CAA = cerebral amyloid angiopathy; FLAIR = fluid-attenuated inversion recovery.

CSF enhancements are, as previously published, defined as hyperintense signal in the subarachnoid space on postcontrast FLAIR, not visible on precontrast images.^24^ Focal and sulcal CSF enhancements are differentiated based on size and shape, with focal CSF enhancement being a punctate hyperintensity, whereas sulcal CSF enhancement is a hyperintensity that fills up the entire sulcus. Figure 1 shows an example of focal and sulcal CSF enhancement. Precontrast and postcontrast FLAIR images were visually compared in MeVisLab (version 3.7.2.12) to rate focal and sulcal CSF enhancements, supported by a calculated difference image (i.e., the postcontrast minus registered precontrast FLAIR image). Enhancement was rated in both the sagittal and transversal views, and the cerebellum and brainstem were omitted. All scans were rated by 3 raters, H.v.d.B., M.K., and S.v.V. The interrater reliability was good (eTable 2), after which a consensus meeting with all 3 raters was held to obtain a final count for focal and sulcal CSF enhancements.

#### DCE Image Analysis

Gadolinium shortens the T1 relaxation time, thus changing signal intensity of the T1-weighted DCE acquisition, which forms the basis of the leakage quantification. This signal is influenced by distortions of the B1+ field and the local T1. Details on correction for B1+ field inhomogeneities and T1 mapping are provided in the eMethods.

The DCE volumes were realigned using MCFLIRT.^25^ On the volume with the highest contrast (∼sixth volume), a manual segmentation of the superior sagittal sinus was made to determine the vascular input function. The DCE timeseries data were fitted with the SEPAL package.^26^ The first 5 volumes after contrast injection were omitted from the fitting to ensure a more steady-state condition for accurate modeling of tracer kinetics. The average of the next 5 volumes of the DCE time series were used as the baseline signal. Signals were averaged over the regions of interest, after which gadolinium concentration curves were generated, while correcting for the adjusted T1 map. The signals were fitted to the Patlak model (deemed the best model for subtle leakage^27^) using linear fitting to generate the quantitative leakage estimate; PS.

Regions of interest were generated based on the segmentation of the T1-weighted scan with SynthSeg version 2.0 from FreeSurfer.^21^ The labeled segmentation was registered to DCE space with affine transformation with FLIRT from FSL.^28^ All segmentations and registrations were visually checked. A whole-brain mask was generated by taking the segmentations and subtracting the CSF, ventricles, brainstem, and cerebellum. The mask was eroded with a sphere kernel with a radius equal to 2 voxels.^29^ A mask of the cortex was generated by multiplying the cortex label from the SynthSeg segmentation with the whole-brain mask, so that only the outer part that borders the CSF was eroded from the mask. The white matter mask was generated by taking the white matter label from SynthSeg and eroding the mask with a sphere kernel with a radius equal to 1 voxel. The normal appearing white matter (NAWM) mask was made by subtracting WMHs from the total white matter mask. We fitted the average signal per region of interest.

### Statistical Analysis

Differences in characteristics for patients with CAA vs non-CAA controls were tested with independent-samples *t* tests for continuous normally distributed data, Mann-Whitney *U* tests for nonnormally distributed continuous data, and χ^2^ for categorical data. A possible association of leakage measures with age and sex was explored with linear and logistic regressions and χ^2^ for categorical data.

In primary analyses, group differences in leakage measures were tested. The difference between patients with CAA and non-CAA controls in the presence of focal and sulcal CSF enhancement was tested with an age-corrected logistic regression, and the differences in CSF enhancement counts were tested with analysis of covariance with an age correction. Group differences in PS in the cortex, total white matter, and NAWM were tested with independent-samples *t* tests. Given the limited sample size, a post hoc power analysis was performed with G*Power (version 3.1.9.7).

In secondary analyses, the association of leakage measures with CMB counts and cSS was tested in patients with CAA. The association of focal and sulcal CSF enhancement presence with CMB count (logistic regression), cSS presence (logistic regression), and cSS volume (tweedie generalized linear model, because of numerous zeros in the dependent variable; cSS volume) was tested with age as a covariate. Among patients with CAA with CSF enhancements, the association between focal and sulcal CSF enhancement count and CMB count (linear regression), cSS presence (logistic regression), and cSS volume (tweedie generalized linear model) was tested with age as a covariate. The association between PS in the cortex with CMB count was tested with a linear regression and the association with cSS volume was tested with a tweedie generalized linear model, without corrections. The association between PS in the white matter and NAWM with WMH volume was tested with linear regression.

All statistical analyses were performed in R version 4.3.2, and the significance level was set at *p* < 0.05.

### Data Availability

The data that support the findings of this study are available on reasonable request from the corresponding author. The data are not publicly available because of privacy restrictions.

## Results

Participant characteristics are shown in Table 1. Patients with CAA and non-CAA controls were well matched for age and cognitive status. As expected, patients with CAA had more CMBs and a higher cSS volume on MRI. Non-CAA controls had subjective cognitive impairment (N = 4) or MCI (N = 3).

**Table 1:** Characteristics of Patients With Probable CAA and Non-CAA Controls

	CAA (N = 14)	Non-CAA control (N = 7)	*p* Value
Age, y, mean ± SD	67.7 ± 9.0	70.1 ± 6.5	0.49
Female sex, n (%)	8 (57)	2 (29)	0.44
MMSE^a^, median (IQR)	27 (26–28)	30 (26–30)	0.26
eGFR^b^, mL/min/1.73 mm^2^, mean ± SD	80.1 ± 19.6	79.3 ± 16.4	0.94
Lobar CMBs, median (IQR)	126 (38–229)	0 (0–0)	<0.001
Deep CMBs, median (IQR)	0 (0–0)	0 (0–0)	—
cSS presence, n (%)	6 (43)	0 (0)	0.12
cSS volume^c^, median (IQR)	0 (0–0.08)	0 (0–0)	0.06
WMH multispot presence, n (%)	9 (64)	2 (29)	0.28
WMH volume^c^, median (IQR)	0.44 (0.31–1.64)	0.20 (0.14–1.25)	0.31
Cortical cerebral microinfarcts, median (IQR)	0 (0–0.75)	0 (0–0)	0.14
Recent small subcortical infarcts, mean ± SD	0 ± 0	0 ± 0	—
PVS centrum semiovale, median (IQR)	2 (1.25–3)	2 (1–2.5)	0.41
PVS basal ganglia, median (IQR)	1 (1–1)	1 (1–2.5)	0.22

Abbreviations: CMB = cerebral microbleed; cSS = cortical superficial siderosis; eGFR = estimated glomerular filtration rate; IQR = interquartile range Q1–Q3; MMSE = mini-mental state examination; PVS = perivascular spaces; WMH = white matter hyperintensity.

a MMSE scores were included from data of the parent study, but only for scores that were acquired within 6 months of participation in the substudy. This resulted in missing MMSE scores for 3 patients and 3 controls. There was no difference between patients and controls in the time interval between acquisition of the MMSE scores and participation in the substudy.

b Missing eGFR data due to missing values after blood draw for 5 patients and 1 control.

c cSS volume and WMH volume are corrected for whole brain volume and expressed as percentage of total brain volume.

### CSF Enhancement: Leakage From Leptomeningeal Vessels

The presence of focal CSF enhancement, but not sulcal CSF enhancement, was associated with higher age (focal CSF enhancement presence: β = 0.49, *p* = 0.02; sulcal CSF enhancement presence: β = 0.37, *p* = 0.10). There was no association between the presence of focal and sulcal CSF enhancement and sex (*p* = 0.84 and *p* = 0.55). Therefore, we decided to correct the group comparisons for age only. Focal CSF enhancement was observed similarly often in patients with CAA and non-CAA controls, whereas sulcal CSF enhancement was seen more often (and only) in patients with CAA (Table 2). All following correlational analyses were only performed in patients with CAA. In patients with CAA, focal CSF enhancement presence or count was not associated with CMB count or cSS presence (data not shown, *p* > 0.10), but in patients with CAA with focal CSF enhancements, focal CSF enhancement count was associated with cSS volume (*B* = 2.61, *p* = 0.003). Sulcal CSF enhancement presence and count did not associate with CMB count or cSS presence (*p* > 0.11), but sulcal CSF enhancement presence was associated with cSS volume (*B* = 4.67, *p* = 0.02). In patients with CAA with sulcal CSF enhancements, sulcal CSF enhancement count was associated with cSS volume (*B* = 1.02, *p* = 0.02). These linear regression analyses were all corrected for age. Of note, owing to time constraints, for 2 participants, the postcontrast FLAIR scans were acquired at ∼15 minutes after gadolinium injection, rather than ∼22 minutes. This may have affected the postcontrast CSF enhancement ratings. Both participants did not have any CSF enhancements on the postcontrast scan; however, because 1 participant was a patient with CAA and the other a non-CAA control, this protocol deviation is unlikely to have affected the group differences.

**Table 2:** CSF Enhancement and PS in Patients With Probable CAA vs Non-CAA Controls

CSF enhancement^a^	CAA (N = 14)	Non-CAA control (N = 7)	*p* Value	Adjusted *R*^2^
Focal CSF enhancement presence, n (%)	7 (50)	4 (57)	0.98	0.16
Focal CSF enhancement count, median (IQR)	4 (2.5–5)	2 (1–3.25)	0.48	0.32
Sulcal CSF enhancement presence, n (%)	5 (36)	0 (0)	0.03	0.27
Sulcal CSF enhancement count, median (IQR)	3 (1–5)	0 (0–0)	—	—

PS (×10^−4^ min^−1^)	CAA (N = 13)	Non-CAA control (N = 7)	*p* Value	Cohen *d*
Cortex, mean ± SD	5.08 ± 4.02	1.29 ± 4.08	0.07	0.94
White matter, mean ± SD	2.60 ± 3.96	0.41 ± 4.57	0.31	0.51
NAWM, mean ± SD	2.54 ± 3.97	0.39 ± 4.62	0.32	0.50

Abbreviations: IQR = interquartile range Q1–Q3; NAWM = normal appearing white matter; PS = permeability-surface area product.

a Focal CSF enhancement related with age (not sex). Therefore, these group comparisons were corrected for age.

**Figure 2:**
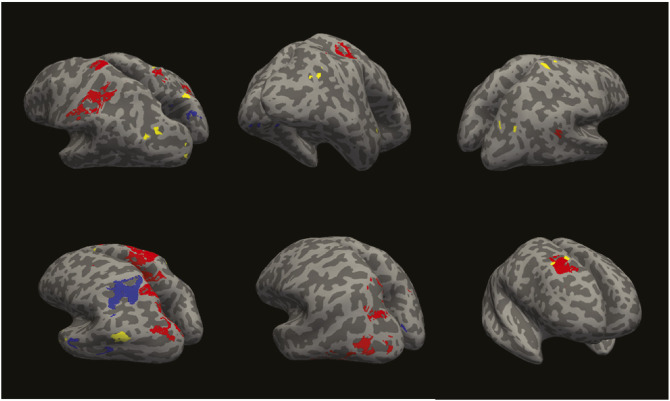
Inflated Cortical Surfaces With Inflated Focal CSF Enhancement (Yellow), Inflated Sulcal CSF Enhancement (Blue), and cSS (Red) Segmentations for Each of the 6 Patients With Probable CAA That Had These Phenomena As exemplified, there is 1 case where 1 focal CSF enhancement directly overlaps an area of cSS. CAA = cerebral amyloid angiopathy; cSS = cortical superficial siderosis.

Given the strong association between focal and sulcal CSF enhancement counts and cSS volume, we explored their relative spatial distribution. Six patients with CAA with focal CSF enhancement and 4 patients with sulcal CSF enhancement also had cSS. These patients together had 26 focal CSF enhancements, of which 1 overlapped with cSS, and 14 areas of sulcal CSF enhancements, of which none directly overlapped with areas of cSS. Figure 2 shows 3D examples for the 6 patients with CAA to show the spatial distribution of the CSF enhancement and cSS.

In an exploratory analysis, we tracked the evolution of CSF enhancements over time at 3, 6, and 24 hours after contrast injection. Eight patients with CAA and 2 non-CAA controls underwent these optional follow-up scans, of whom 4 patients and 1 control had CSF enhancement on the original postcontrast T2-FLAIR. A total of 12 focal and 4 sulcal CSF enhancements could be tracked over time. A qualitative assessment showed that enhancements were still visible at 3 and 6 hours postcontrast, but not at 24 hours. See eFigure 1 for a representative example and eTable 3 for more details on the specific signal changes over time. No new CSF enhancements were visible at 3, 6, or 24 hours.

### Permeability-Surface Area Product: Leakage From Small Parenchymal Vessels

**Figure 3:**
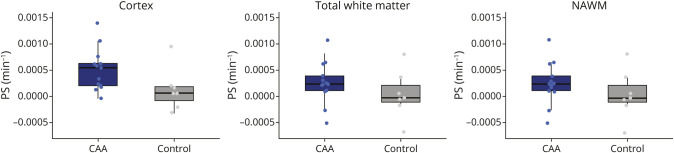
PS in the Cortex, Total White Matter, and NAWM in Patients With Probable CAA vs Non-CAA Controls CAA = cerebral amyloid angiopathy; NAWM = normal appearing white matter; PS = permeability-surface area product.

Owing to a technical failure, 1 DCE scan failed, leaving 13 patients with CAA and 7 non-CAA controls for the PS comparisons. PS was not associated with age or sex (*p* > 0.51). We therefore did not correct the group comparisons. PS in the cortex was nonsignificantly higher in patients with CAA than in non-CAA controls (Table 2, Figure 3) with a large effect size (Cohen *d* = 0.9). Within patients with CAA, PS in the cortex was not associated with CMB count (*p* = 0.70), cSS presence (*p* = 0.14), or cSS volume (*p* = 0.49). PS in the white matter and NAWM were similar in patients with CAA and non-CAA controls (Table 2, Figure 3) and were not significantly associated with WMH volume (*p* = 0.10 and *p* = 0.12, respectively).

## Discussion

This exploratory study aimed to image BBB leakage in vivo in CAA by measuring extravasation of gadolinium-based contrast agent with 2 complementary imaging sequences. We found greater sulcal CSF enhancement in patients with CAA than in non-CAA controls, which was associated with higher cSS volume. Smaller, focal CSF enhancements were seen similarly often in patients with CAA and non-CAA controls and were associated with higher age, as well as with cSS volume in the patients with CAA. Nonsignificant but numerically higher PS was observed in the cortex in patients with CAA, with between-group differences likely underestimated given the comparison with a control group with subjective cognitive complaints or MCI. PS was not associated with CMB count or cSS volume.

CSF enhancement on FLAIR imaging after injection of gadolinium-based contrast agent reflects extravasation of the contrast agent across the BBB into the CSF space, where it shortens the T1-relaxation time, resulting in a hyperintense signal.^11,24^ It is therefore thought to represent BBB leakage at the level of the leptomeningeal vessels.^11^ We distinguished between focal and sulcal CSF enhancements. Focal CSF enhancements were often observed in patients with CAA and non-CAA controls, occurring in approximately half of all participants. Their presence and count were associated with higher age, which is a relation that has been reported before in the literature and suggests that subtle leakage of the BBB occurs with normal aging as well.^11^ Sulcal CSF enhancements, on the other hand, were only seen in patients with CAA. Importantly, in patients with CAA, both focal and sulcal CSF enhancement counts were associated with cSS volume. Therefore, it seems plausible that sulcal CSF enhancements are etiologically similar to focal CSF enhancements. There is one previous study that also described CSF enhancements in patients with sporadic and Dutch-type CAA,^30^ although without the administration of a contrast agent. They detected endogenous enhancements on 7T FLAIR imaging, hypothesized to be a sign of extravasation of plasma proteins and/or erythrocytes.^30^ Although this study did not explicitly differentiate between focal and sulcal endogenous CSF enhancements, the examples provided resemble sulcal CSF enhancements. The authors found more endogenous CSF enhancements in patients with CAA than in non-CAA controls and also report a strong association with cSS.^30^ Interestingly, CSF enhancements often—but not always—directly spatially overlapped with cSS, which is something we did not observe in this study. Although it is not certain that the endogenous CSF enhancements on 7T and the postcontrast CSF enhancements from this study represent similar underlying phenomena, given the very similar observations, this seems a likely possibility. Importantly, CSF enhancements have also been reported in other neurologic conditions, such as ischemic stroke and Alzheimer disease.^11,24^ CSF enhancements thus are not specific to CAA pathology, but the strong association between CSF enhancements and cSS warrants future studies into the prognostic value of CSF enhancements in the context of CAA. Given that CSF enhancements are thought to be a sign of BBB leakage of the leptomeningeal vessels and that cSS is thought to be indicative of leakage of blood from these same vessels, the hypothesis arises that CSF enhancements may be a more subtle form of leakage that could potentially precede cSS. Because cSS is an important predictor of ICH, the most impactful clinical manifestation of CAA,^31^ the relation between CSF enhancements and cSS is likely to be clinically relevant and should be further addressed.

PS is thought to quantify BBB leakage from the small parenchymal vessels, mostly the capillaries.^32^ Although not statistically significant (likely due to limited power), leakage from small vessels in the cortex seemed higher in patients with CAA than in non-CAA controls, whereas there were no indications for differences of PS in white matter small vessels. Given the site of vascular Aβ accumulation—the cortex—these findings indicate that in CAA, in vivo measurements of PS in the cortex may be directly associated with underlying CAA pathology. The local PS increase in the cortex is thus likely CAA specific. This is supported by the characteristics of the non-CAA control group that consisted of patients who also suffered from subjective cognitive complaints or MCI. Although no diagnostic information was available to determine underlying pathologies in these control cases, Alzheimer disease and vascular pathology are the most common contributors to cognitive impairment. Indeed, control participants had signs of cerebral small vessel disease–related manifestations, including extensive WMHs. In previous research, Alzheimer disease and cerebral small vessel disease have also been related to BBB leakage,^12,33^ which means that the differences observed in this study are likely to reflect changes specific to CAA, rather than the general features of cognitive impairment. Unexpectedly, no association with CMBs or cSS volume was found in this study. Whether this null finding is due to insufficient power or reflects the absence of a direct association requires investigation in larger studies. A different approach to further explore the relation between leakage and disease severity would be to study leakage in the occipital vs the frontal lobe, to determine whether leakage follows the posterior-to-anterior distribution that is typically observed in vascular Aβ pathology and (micro)hemorrhage burden. Unfortunately, the data did not allow for such a comparison because of distinct noise patterns in these brain regions, which hindered a reliable assessment.

Further in vivo support for leakage through the BBB in CAA comes from studies that have used contrast-enhanced MRI to perform vessel wall imaging and reported vessel wall enhancements in patients with CAA.^34,35^ Interestingly, a small recent study in patients with CAA-related inflammation reported that such small parenchymal enhancements preceded CMB formation.^36^ These enhancements on vessel wall imaging are often interpreted as a sign of inflammation but could also be interpreted as leakage of the BBB, which subsequently triggers an inflammatory response.

An important strength of this study is that we were able to assess BBB leakage in complementary vessel populations that are known to be primarily affected by CAA pathology, which may detect different but biologically related patterns of BBB dysfunction driven by the underlying heterogeneous expression of CAA pathology in these vessel populations. Another strength is the selection of patients with CAA without a history of ICH. The fact that BBB leakage can be detected in vivo in this group warrants further studies to explore whether these measures could serve as predictive or outcome markers in clinical trials, in a disease stage where symptomatic ICH could possibly still be prevented.

The main limitation of this study is the small sample size. Regardless, we found significant group differences in sulcal CSF enhancement and observed a significant association of CSF enhancements with cSS. For PS, a measure with a lower signal-to-noise ratio,^29^ the small group sizes likely were an impediment. Despite the large effect sizes, a power analysis showed that this study had a low power of 47% to find significant group differences in PS in the cortex. To reach 80% power, group sizes of 19 participants would have been required. Owing to logistical constraints and an MRI scanner upgrade, we were unable to include more participants, and therefore, this study should be considered exploratory. In addition, ex vivo BBB leakage is often studied by assessing extravasation of blood products, such as hemoglobin, fibrinogen, and IgG. Given the small molecular weight of Dotarem (=0.75 kDa^37^), we cannot be sure that the reported findings also translate to leakage of larger molecules. Dotarem is, however, considered one of the safer contrast agents,^38^ justifying its use in this study. Finally, not many previous studies have assessed PS in the cortex because of concerns about partial volume effects from the CSF, especially in a population with possible cortical atrophy. We tried to mitigate partial volume concerns by eroding and carefully checking the cortical masks. Although it is a significant step that BBB leakage can be visualized in vivo, the true value would lie in it being a pathology specific marker that can predict disease progression and could serve as an outcome measure in clinical trials. Whether leakage of the BBB is indeed an early marker of CAA that predicts, or possibly directly causes, clinical symptom progression, cannot be determined from this study. The findings from this explorative study indicate that future larger studies with follow-up data to address these questions are warranted.

In conclusion, BBB leakage in the form of gadolinium-based contrast agent extravasation can be measured in vivo in patients with CAA without a history of lobar ICH. Sulcal CSF enhancement, reflecting leakage from leptomeningeal vessels, was seen only in patients with CAA, not in non-CAA controls with subjective cognitive complaints or MCI. CSF enhancements were associated with higher cSS volume. Numerically higher PS was seen in the cortex in patients with CAA vs non-CAA controls with subjective cognitive complaints or MCI. Future studies with follow-up data are required to determine where the observed leakage fits in the pathophysiologic framework of CAA and to see whether it could serve as a predictive and/or therapeutic outcome biomarker.

## Glossary

Aβ: β-amyloid
BBB: blood-brain barrier
CAA: cerebral amyloid angiopathy
CMB: cerebral microbleed
cSS: cortical superficial siderosis
DCE: dynamic contrast-enhanced
DWI: diffusion-weighted imaging
eGFR: estimated glomerular filtration rate
FLAIR: fluid-attenuated inversion recovery
ICH: intracerebral hemorrhage
IgG: immunoglobin G
MCI: mild cognitive impairment
NAWM: normal appearing white matter
PS: permeability-surface area product
SWI: susceptibility weighted imaging
WMH: white matter hyperintensity
